# Abstracts and Gleanings

**Published:** 1888-02

**Authors:** 


### ABSTRACTS AND GLEANINGS.

**The Enemata Syringe—Some Suggestions as to its Use and Value.**—Dr. J. W. Milam (in *Indiana Medical Journal*) very sensibly remarks: "An article on 'the syringe' would cover a wide field. Even discarding the hypodermic and fountain syringes—yea, even the piston and all other forms of syringes, and considering only what is commonly called the Davidson or bulb syringe, would give material for a very long essay. The present purpose is simply to advocate the more common use of the last-named syringe in giving injections into the rectum. The use of the hypodermic syringe need not be urged upon the intelligent practitioner. The p. p male syringe has *more* than its full share of popular and professional favor. Probably most physicians are prompt enough in washing out bladders and vaginas, and even uteri, but my observation teaches me to believe that the profession do not give enemata as often as might be done with profit. In this brief paper I do not propose to discuss the subject of rectal feeding. Except in those rare cases of local disease of the alimentary tract in which it is impossible to feed by means of the stomach—only in those cases do I believe in resorting to nutrient enemata. We have learned from Dr. Tanner and his disciples, as well as by subsequent scientific experiment, that we used to be *foolishly* solicitous about feeding our patients. Hence, I remark that I do not think rectal feeding needs any urging from my pen. The two most frequent uses to which I put the bulb syringe are injections of water and injections of medicines into the rectum. As to the latter use, it is, in the majority of cases, for local trouble. When persistent vomiting precludes the use of the stomach, however, frequent resort is had to rectal injections of the ordinary medicines (generally in perfect solution, of course). Certainly when the drug can safely be given hypodermically, that is preferable, on the grounds of convenience, rapidity of absorption, and definiteness of result. In chronic constipation we all aim, among other things, to establish a definite habit of going to stool at a given hour each day. And rightly enough too. Why, I have seen cases, of only a few months standing, cured by giving a nightly dose of compound cathartic pills, and am sure the resulting *habit* of rising early for a definite and *pressing* reason had more to do with the cures than any permanent alteration in the disordered organic function. How much better, then, to establish this habit by use of daily enema of warm water, or mild, weak soap-suds. The theory is good, and the results from a few trials are more than satisfactory. Then there is more than this in the procedure. The daily contact of warm water with the mucous membrane of the rectum and colon determines some extra amount of blood to the part, causing increased glandular activity and improved general tone. Surely cleansing and dilating the lower two or three feet of the bowels ought to be salutary. As to the addition of castor and other oils to the water or soap-suds, I have entirely abandoned it. It is troublesom, it gums up the valves of the instrument, and it is unnecessary generally at least. I do sometimes add a little molasses (sorghum preferred), as it dissolves in the water, giving no inconvenience, and has a stimulating influence on the mucous surface. Do I not use medicines in conjunctions with this treatment in chronic constipation? Yes, I think it best always to do so, although I have tried the enemata alone in two cases, for a short time—successfully in one case, and with excellent results in the other.

As an aid in relieving a patient of an impaction of fæces, the syringe is invaluable. It is also of great service in conducing to an easier evacuation in almost all cases of recent or "acute" constpation. In hemorrhoids it is a boon to the patient. All physicians know that there are many people afflicted with piles who will not submit to the proper treatment, whose daily evacuations are their daily dread, in anticipation, and daily agony in realization. From experiance, I *know* that not only can life be made more bearable to them, but that from one pint to one quart of warm water will soften the fæces, relieve the straining and consequent congestion, and in recent cases will cure the hemorrhoids without any conjoined treatment. In extreamly sensitive cases a preliminary injection of a two per cent. solution of cocaine should be given with a small piston syringe.

In my opinion the most important use of the injections of fluid into the rectum is one not yet mentioned in these rambling remarks. I claim little originality in anything mentioned, but my experience in the use of enemata of cold water, and of various medicaments in the treatment of acute dysentery, has been so gratifying that I can not close this article without mention of it. In some cases a cure can be accomplished by one or two injections of a pint of very cold water. In ordinary cases of dysentery, if the symptoms are not severe, my usual treatment is cold water injections every four to six hours, and a small powder of podophyllin, leptandrin and hydrag. cum creta, given about the time of each injection, until a copious biliary stool is produced. Then a few doses of bismuth and Dover powders. In some of the cases attended by severe tenesmus I give injections of cocaine solution. In others laudanum and starch-water. Again, the cold water injections can be beneficially alternated with injections of a half teacupful of sweet oil, in which has been shaken up a little laudanum, tinct. quassia and subnit. bismuth.

Much more could be said in favor of a more extended use of the syringe, but my object is to call the attention of those who use it in the rectum, but seldom to its varied utility. I am aware that many in the profession will find but little that is new in these remarks, but the space here used is not wasted if the doctors, both old and young, who seldom use it, will read and consider the claims here urged. Furthermore, it seems to me that it would be profitable if the Editor would open these columns to a discussion of the uses and abuses of the syringe by various contributors.

**Amylene Hydrate.**—The average dose of this new hypnotic is 40 to 60 grains, best given in red wine or in extract of liquorce: "No disagreeable after-effects were noted, no disturbance of the appetite or in the action of the bowels, and but rarely slight headache on the following day. Of special importance is the fact that the statement of Von Mering is confirmed, that amylene hydrate produced no modification of the respiratory or circulatory organs. The rhythm and number of the respiratory movements are not to be distinguished from those of normal sleep. No change in the number of the pulsations or in the pulse-curve, as examined by the sphygmograph, were detected after the administration of the amylene hydrate; in some cases a slight increase and in others a slight decrease in the pulse frequency being scarcely worthy of notice, and presenting no greater variety than might be expected between the sleeping and the waking states. As yet no positive statement can be made as to whether patients become accustomed to this hypnotic and its effects gradually wear off, but it would seem that its having been used constantly with success in two cases for thirty-nine days would warrant a negative answer. Of the eighty cases in which this drug was employed, twelve consisted of melancholia with stupor, five of melancholia agitata, thirteen of dementia paralytica, one of epilepsy with alcoholic dementia, four of primary dementia, seven of hallucinatory insanity, one of hypochondrical insanity, one of stupor, one ot chronic alcoholism with insomnia, ten of mania, two of delirium, four of delirium acutum, six of delirium tremens, one of hysteria with insomina and delirium, one of epilepsy with excitement and delirium. It would appear from these results that the tertiary amyl alcohol has proved itself to be a reliable hypnotic, since it can be employed in heart affections, and is without influence on the alimentary canal. It is superior to urethan, since it produces sleep even in states of the wildest excitement, and is at any rate equally as good as paraldehyde in efficiency, and is free from the disagreeable odor and taste of the latter."—*Therapeutic Gazette.*

**Strophanthus.**—This new African heart-drug continues to attract attention both abroad and in this country, and the evidence seems to be sufficient to show that it has great practical value.

Professor Drasche (*Wien Med. Blatter*, 1887, page 586) found 5 drops of the tincture of strophanthus given to the healthy man would produce in about three hours a fall of from about 8 to 12 beats per minute of the pulse, which would last part of the day. After 10 drops the pulse fell in half an hour 12 to 20 beats; after 20 drops the pulse sank in the single case given from 84 to 54 strokes. There was no influence on the respiration, but a lowering of the temperature, in some cases as much as a degree. Fifteen drops given hypodermically caused great irritation and pain and swelling at the place of injection, violent headache, repeated vomiting and nausea, and copious secretion of urine: the pulse fell in an hour from 96 to 84, and the next day the headache and general ill feeling continued, with a very pronounced local irritation. In a strong forty-five-year-old man suffering from left-sided pneumonia, four doses a day of 5 drops of tincture of strophanthus was followed in twenty-four hours by a fall of the pulse 18 beats per minute, and by a distinct slowing of the respiration and reduction of the temperature 0.8° C. The pneumonia itself was not affected, and the crisis came on the ninth day. In another similar case, 40 drops of the tincture a day was followed by marked fall of the pulse and increase of the urine. Strophanthin was later given in solution, the four doses each amounting to 2 milligrammes. The pulse became very irregular, varying from thirty-six to sixty beats every minute. There was marked increase in urinary secretion and great lessening of the dyspnœa. In a case of aortic insufficiency, which came into the hospital almost moribund, with the pulse ninety-six, scarcely to be felt, 15 drops in an hour caused the pulse to fall to seventy-two and become much stronger, and the patient slept through the night, for the first time in three months. Afterwards 30 drops of strophanthus tincture being given daily, the pulse fell to sixty; but the patient complained of so much burning in the œsophagus and stomach and nausea that the medicine was withdrawn and digitalis substituted. It, however, failed to have influence, and the man shortly afterwards died suddenly. In a second case of aortic insufficiency, the tincture of strophanthus was given for four weeks regularly, 40 drops a day, with great relief to the dyspnœa. Besides these cases, Dr. Drasche reports several others in which the strophanthus tincture produced results similar to those that have just been described. The effect upon the excretion of urine seems to have been most extraordinary. In some instances the amount excreted after the administration of the drug was five, or even six, times what it had been before. Almost universally when the strophanthus was pushed the patient complained of burning in the œsphagus and stomach, with loss of appetite and extreme gastric distress, which not rarely rose to vomiting; sometimes there was diarrhœa. In no case was there any evidence whatever of cumulative action. The action of the drug is not only more prompt in coming on, but is evidently less permanent than is that of digitalis.—*Therapeutic Gazette.*

**Methylene Chloride Compared with Chloroform.**—(*Ther. Gazette*) Their experiments in comparing the action of chloroform and pure methylene chloride may be stated in a few words, as follows: First, narcocis occurs with both remedies after about the same time. Second, with both drugs there is a preliminary period of excitement. Third, with methylene chloride there is no increase of pulse in the period of excitement; there is in the course of chloroform. Fourth, in both cases after the occurrence of the narcosis there is slowing of the pulse and respiration, though these symptoms occur sooner and to a greater degree in the chloroform narcosis, and the complete arrest of the pulse and respiration occurred much sooner in the case of chloroform than with methylene chloride. The reduction of temperature in narcosis is with both about the same. Methylene chloride produced salivation; chloroform does not; methylene chloride produced transient narrowing of the pupil. In methylene chloride narcosis the rigidity of the neck is more marked than in the case of chloroform, but convulsions of the limbs are more frequently seen with chloroform than with methylene chloride. With these points of similarity, the only advantage which might be attributed to the methylene chloride is its very much less dangerous action on the heart. For while in chloroform narcosis after the administration of only 5 cubic centimetres the heart-action is often imperceptible, the authors claim that they have frequently produced profound methylene chloride narcosis for nearly an hour without ever producing any unfavorable action on the heart. They also state that even when the pulse becomes arrested by the methylene chloride the animals are much more readily and rapidly resuscitated through artificial respiration. In comparing the narcosis produced by pure methylene chloride and mixtures of alcohol and chloroform, it was found that the addition of methyl alcohol to chloroform somewhat reduced the poisonousness of the latter without reducing its narcotic action, and therefore is to be preferred to pure chloroform as an anæsthetic. The mixture which they recommend consists of 45 parts of chloroform to 13 of methyl alcohol. They conclude their paper with the following sentences. First, that the substances heretofore employed as methylene chloride is a mixture of chloroform and methyl alcohol. Second, this mixture is to be preferred to chloroform for the production of narcosis. Third, that pure methylene chloride produces narcosis quite as rapidly and as profoundly as either of the above preparations. Fourth, that the action of pure methylene chloride on the circulation and respiration is, by far, less dangerous than that of either of the other preparations.

**Operative Treatment of Internal Obstruction of the Bowels.**—The treatment of persistent obstruction of the bowels, dependent upon some intra-abdominal condition, is one of the most interesting questions in medicine or surgery. There are able and conscientious surgeons who think the tendency at the present time is too much toward early operative interference. Prof. John Ashhurst, of this city, for example, and Prof. Madelung, Rostock, have presented strong reasons for prudence in proceeding to operation. On the other hand, the comparative safety with which the peritoneal cavity may be opened, in these days, has induced other surgeons to protest that many patients lose their lives because they are not given the chances of an operation.

At the last Congress of German Surgeons, there was an active discussion of this subject, and the general opinion was favorable to operation in cases which do not readily yield to medicinial treatment, and in which there is a strong presumption of a mechanical condition not amenable to medicinal measures.

As to the operation to be chosen, the majority of those who spoke favored the formation of an artificial anus. But Kummel, of Hamburg, in the *Centralblatt fur Chirurgie*, November 5, 1887, opposes this opinion, and gives the reasons which incline him to choose laparo omy as the operation best suited for internal obstruction of the bowels. In support of his views, he cites his own experience in ten cases, in seven of which he performed a laparotomy, and deplores the delay of medical men to submit their patients to surgeons until after they have exhausted all other means, and their patients are *in extremis*. He mentions a case in which he himself abstained from operation because he attributed his patient's trouble to tubercular peritonitis—for which he had good reason—and found at the autopsy that the symptoms of obstruction were due to a band compressing the upper part of the small intestine. The peritonitis which exists in many cases of obstruction, and which is sometimes regarded as its cause, he believes to be usually secondary to it. Taking everything into consideration, he pronounces strongly in favor of laparotomy when the circumstances justify an operation, and thinks this furnishes the best way to discover the exact nature of the obstruction and the best means of overcoming it. To make an artificial anus is to lose the advantage of sight and touch in determining the condition on which the obstruction depends, and to do an operation sometimes ineffectual and often demanding another operation by no means free from danger.

The paper of Kummel, to which we have referred, is too long for us to do it justice here, but we cannot refrain from giving our readers the benefit of the author's suggestions in regard to a part of the operation of laparotomy, which is often very difficult, namely, the reposition of the intestines. Kummel's method of accomplishing this is to place a folded napkin or towel over the bowels, and to tuck its edges of the incision, upwards under the sternum, downwards into the pelvis, and on the sides under the abdominal wall. When this is done, and the whole is controlled by the hands of an assistant, he places the approximating sutures in position and has the towel withdrawn gradually as the sutures are tightened. In this way he says he has no difficulty in getting the bowels in place, and no need for the dangerous procedure of puncturing them so as to empty them of gas. He mentions, also, with approval, the suggestion of Rehn to empty the bowels of gas by introducing a stomach tube into the stomach.

We commend these views of Kummel to our readers, for we share in his conviction that laparotomy for internal obstruction is a justifiable operation, and that long delay in undertaking it is more dangerous than the operation itself, if good judgment and skill are used in its performance.—*Medical and Surgical Reporter.*

**Venesection in Pneumonia.**—Dr. Meacham (in *Medical Standard*) writes: "Treatment of pneumonia has undergone many changes since my advent into the profession, and I doubt very much whether more cases are saved by this change. I feel sure that more subjects pass on to the second and third stages than was the case under more heroic treatment twenty-five or thirty years ago. Non-use of the lancet is a great mistake in many cases. I firmly believe the day is not far distant when the practice of bleeding in pneumonia will be revived, not as extensively and in all stages of the disease as formerly, but much more frequently than for many years past. I believe that no remedy compares in effectiveness with a liberal bleeding in the very early stage of this disease, if the patient be of fair vigor. I desire to emphasize *early*. After the lungs have been filled by infiltration and weigh three or four times more than is natural, then by no means bleed, for the system has already been depleted too much, and the product thrown where it is least wanted. There are times and seasons when I would not wish to be understood as advocating bleeding, as when and where typhoid and other adynamic diseases prevail. Very young persons and very old ones, as a rule, may be left out of the list, though I have resorted to venesection in both a few times in my life, with splendid results.

In the congestive and early stages of pneumonia every vessel of the affected portion is filled with blood to its utmost extent, and a remedy that will relieve that pressure suddenly and effectively (other things being equal) is the one needed. Venesection is that remedy, and after its use other means can be employed with more rapid and lasting action."

**Intestinal Obstructions.**—Dr. N. Senn, Milwaukee ("International Medical Congress Proceedings"), says that for resection and circular enterorraphy, a fistulous communication, between the bowel above and below the obstruction, should be substituted in all cases where obstruction is not an intrinsic danger. Gastro-enterostomy and jejuno ileostomy must be done by approximati on with decalcified perforated bone-plates. Colon or cæcum anas tomoses can be established by apposition with decalcified perforated bone-plates, or by lateral implantation of the ileum into the colon or rectum. An ileo-colostomy, or ileo-rectostomy by approximation with decalcified perforated bone-plates or lateral implantation, should be done in irriducible ileo-cæcal invaginations where the local signs do not indicate the occurrence of gangrene and perforation. In threatened gangrene and perforation the invaginated portion should be excised, both bowel ends closed, and intestinal canal continuity restored by making an ileo colostomy by approximation with perforated decalcified bone plates, or by lateral implantation. Restoration of intestinal canal continuity by perforated approximation plates. or lateral implantation, should be resorted to in all cases where circular enterorraphy is impossible on account of the difference in the size of the lumina of the two ends. Definitive healing of an intestinal wound is only completed after the formation of a net-work of new vessels in the product of tissue-surfaces. Under favorable circumstances quite firm adhesions are formed between the peritoneal surfaces within six to twelve hours which effectually resist the pressure from within outward.—*Medical Standard.*

**A Case of Poisoning with Cocaine.**—H. Schnydey, in the *Correspondenze-Blatt f. Schw. Aertze*, No. 15, 1887, reports a case of poisoning, which occurred under the following circumstances: A young apothecary, of somewhat nervous habit, was advised by a friend to take $\frac{1}{3}$ of a grain of cocaine, repeated in three-quarters of an hour, for violent headache. As he lost the muscular sense in his extremities, and developed oppression in breathing, he took as an antidote to the poison 20 drops of tincture of nux vomica. After about half an hour, his extremities, which were icy cold, began to tremble violently. His pupils were immovable, moderately dilated, pulse thready and one hundred and fifty to the minute. The action of the heart was strikingly feeble, and in addition there was violent excitement, and at times delirium. Strong coffee was administered, warmth applied to the extremities, and chloroform sprinkled on cotton and inhaled.

In an hour respiration became freer, pulse fuller and one hundred and twenty to the minute. On account of the violent headache, cold applications were made to the head and a mustard plaster to the chest. After four hours had elapsed, the pulse sank to eighty beats, but sleep was not obtained for some time.—*Med. and Surgical Reporter.*

**Galvanism in the Treatment of Indolent Ulcers.**—Attention is again called to this long-known but neglected therapeutic procedure by the report of a case occuring in a man of 20, on whose leg an ulcer had remained unhealed fifteen years. The ulcer, which was situated an inch above the internal malleolus, extending across the leg transversely to the subcutaneous border of the tibia, and internally about two inches, was indolent as to its general characteristics.

Local applications and constitutional treatment never gave relief until he commenced the use of the galvanic battery. The secretions of the ulcer were noticed to be alkaline, and the negative pole was placed to the ulcer, the positive to some indifferent point, and six cells were taken into the circuit; the seance was prolonged about ten minutes. From that time the ulcer began to get well, and a half dozen applications ended the case. He applied the bare silver plate of the negative electrode directly to the diseased surface. The other treatment was also persevered in.—*The Med. Analetic.*

**Cocaine for Neuralgia.**—A writer in *Brittish Med. Journal* in a long standing and obstinate case of facial neuralgia used cocaine as follows: One-sixth of a grain injected into the cheek hypodermically gave on the first day relief for several hours. For three successive days after this $\frac{1}{3}$ of grain gave relief only for an hour or so. After this I injected $\frac{1}{2}$ a grain night and morning with the most completely satisfactory results. After three days at this rate I gradually diminish the dose, and the pain ceased entirely.

Since this occasion, during the last six months, two attacks have begun, but in both cases been stopped at once by the injection of cocaine in $\frac{1}{2}$ grain doses.

During the i tervals of attack no cocaine is ever used, but, armed with a hypodermic syringe and a few of Burrough's and Welcome's $\frac{1}{2}$ gr. tabloids, the patient now goes away for several days at a time on shooting expeditions, confident of being able to "doctor" his old enemy effectually, should he venture an attack while camping away from home.

**Treatment of Rheumatism in the Jefferson College Hospital** —Dr. Da Costa treats his cases of acute rheumatic fever, as a rule, with salicylic acid, about a drachm in twenty-four hours; he does this especially in the cases of active rank character, in which the joint affection is decided. Where marked cardiac complication exists, he greatly prefers the alkaline plan of treatment; indeed, has little faith in the use of salicylic acid either to prevent cardiac complications or to remove them. Nor does he, in any case, continue salicylic acid or the salicylates if no impression is made on the rheumatic malady in three or four days. When the remedy does good at all, his experience is that it does good quickly.

In cases of acute or subacute muscular rheumatism, or in subacute articular rheumatism, with much pain and only moderate swelling of the joints, he often employs bromide of ammonium, or if this fail, nitrate of potassium. He uses opium sparingly, and generally confines it to a moderate dose or two of Dover's powder, given at night

He strongly insists, no matter what plan of treatment be adopted, on the addition of quinine, from 12 to 16 grains daily, as soon as the more active symptoms have subsided, believing that thereby the patient's strength is sustained and relapse prevented.

From tincture of chloride of iron he has seen no good, except in pyemic rheumatism or in kindred forms of rheumatism.

Locally, he does little, enveloping the swollen joints, if very painful, in a thin layer of cotton-wool; where the joints are very much swollen he envelops them in cloth wrung out in a strong solutlon of nitrate of potassium, with morphia added.

The diet is always bland and unstimulating, chiefly milk, farinaceous substances, and very moderate amounts of broths, eggs and fish Alcohol is not given, except in the so-called "typhoid cases," in which also high temperature is generally found.—*Med. News.*

**Tobacco Amblyopia.**—Dr. George Howe, of Columbia, S.C., after reporting two cases (Trans. S. C. Med. Ass'n, 1887), raises the question as to whether there is not a true amblyopia from the use of tobacco Both of them were also drinkers. He quotes numerous authorities to show that it is not so much tobacco that causes amblyopia or amaurosis as it is alcoholic drinks. In Turkey, where smoking is the habit, amaurosis is rare. Minor, to test the matter in some cases, interdicted alcoholic drinks, but allowed his patients to continue the use of tobacco, and they got well. Dr. Howe is disposed to give alcohol the credit of bringing about this impairment of vision rather than tobacco. All his cases have been drinkers as well as smokers. His treatment is abstinence from both, and beginning on 1-32 gr. of sulphate of strychnia, gradually increasing until signs of its physiological action are seen.— *Virginia Medical Monthly.*

"**Doctor,** what do you think of the new treatment for consumption by injecting carbonic acid into the rectum ?"

"Well, it certainly distracts the mind of the patient for awhile, and may do good by its moral effect by making him think of his latter end; he becomes, as it were, conscious of his rectum, as the schoolboy rendered *mens conscia recti*. He may even 'bacilli' enough to cherish the hope that he is getting cured, and is taking a short cut to health over this *pons asinorum*, but I regret to say that I think it will end as it began—in gas."—*Medical World.*

**Amylene Hydrate.**—In all, Mehring has given amylene hydrate to sixty different persons in three hundred and fifty different doses, each dose varying from 50 to 90 drops. The affections in which he employed the substance were of the most varied character—sleeplessness from nervousness, delirium tremens, phthisis, and in convalescence from various febrile diseases. Only in four cases out of all these did it prove inefficacious. Twice it was employed in whooping cough with a favorable result, but like chloral, it proved unreliable in producing sleep when the wakefulness was due to pain. It is said to possess a less disagreeable taste than paraldehyde, and the exhalations after taking it are not so disagreeable. From its freedom from danger and more pleasant taste, it is evidently to be preferred to either chloral or paraldehyde, even though it be weaker, as a hypnotic, than chloral. It may be administered internally or by enemata. In sleeplessness from pain, amylene hydrate may with advantage be combined with hydrochlorate of morphine. No unpleasant after effects of any kind, according to Von Mehring, follow the employment of this substrance; no nausea, vomiting, headache, or disturbance of digestion.

Other hypnotics have from time to time been as highly lauded, but were found to have serious drawbacks. This may not be any better, but its prospects yet appear very promising.—*North Carolina Medical Journal.*

**Sore Nipples.**—A writer in *Medical World* says: "A rational treatment may be summed up thus: In the early stage, when the nipple is simply sensitive and tender, nothing is more likely to prevent ulceration than the following formula:

R. Plumbi nitratis............................gr. x–xx,
   Glycerine..............................f ℨ j.
M.

In the early stage of erosions, I use compound tincture of benzoin, three or four coats, with nipple shields if possible. If ulceration exists, stop nursing from that nipple, empty the breast by gently rubbing only, paint over ulcerated surfaces with nitrate of silver, gr. x to f ℨ j, water, and dust with dry powder.

For cracks or fissures I pencil with nitrate of silver, and then cover with collodion; if uncomplicated they will be easily healed. Should there be inflmmation of the nipples, which is sometimes the cause and often the consequence of excoriation, first use a soft bread and milk poultice and then lead an opium lotion. After the inflammation is so much subdued that nursing can be borne, apply a lotion of the glycerate of tannic acid.

**Decoction of White Oak Bark in Internal Hemorrhoids.**—Dr. Jacob U. Stout, in *Medical Waif*, writes: "In the November issue of *The Waif* I see a request that physicians report their experience in treating rectal diseases. Now I am no specialist, and don't claim to know everything and the price of it, but I do know well, that I have cured many cases of internal piles by nothing more or less than a strong decoction of white oak bark. I do it in this manner. I order a decoction to be made as follows:

℞. White oak bark ............................... ℥ ii,

Water ............................................ O ii.

M. Boil till there is 1 pint, then strain and have the patient inject 3 or 4 ounces night and morning, and retain it for from ten to twenty minutes.

By this means the piles shrivel up and all hemorrhage ceases, and the patient will soon get well. In cases of piles where there is great itching around the anus, this remedy works very quickly, often relieving after two or three injections. This decoction is of much benefit in prolapsus recti, and I venture to say that there is no one remedy that will give you such good results in piles and prolapsus as white oak bark. Try it.

**Rabies from Tanacetic Acid.**—At the meeting of the Academy of Medicine of Paris, October 18, 1887, M. Hayem read a paper by M. Peyraud, on "A Comparative Study of Tanacetic Rabies," (*Concours Medical*, October 22, 1887). In this paper the author contended that the essence of tansy has a more powerful influenc in producing rabies in all animals, except frogs, than true rabies has. The symptoms during life and the lesions found post-mortem are similar. The phenomena are due to excitation of the medulla oblongata, and of the roots of the pneumogastric nerves. (*Progres Medical*, Oct. 22, 1887.) M. Colin, who knows a great deal about rabies, questioned the propriety of giving the name of rabies to the symptoms produced by tansy. In this he was, no doubt, right; but, as the diagnosis of rabies rests upon certain symptoms, which are held to be characteristic when they are seen in the subjects of other experiments, it is hard to see how their significance can be denied by some members of the French Academy in those of M. Peyraud.—*Medical and Surgical Reporter.*

**The Texas Supreme Court** has recently decided that criminal assault committed while the female is under the influence of chloroform does not constitute rape.

This is in opposition to many English and most American decisions, although the same view is held by some English judges. The whole question turns on the Texas legal definition of rape. The Texas medical journals which have denounced the Supreme Court for its decision should remember that it has been repeatedly demonstrated that chloroform cannot be administered by a single person unless by consent, and furthermore that delusions of rape are engendered by chloroform. Some innocent physicians will be saved from dishonor by the precedent thus set by the Texas Supreme Court. Where there is a doubt, the court is fully justified in deciding it in favor of inocence.—*Medical Standard.*

**Treatment of Dysentery in Texas.**—The *Texas Courier Record of Medicine* gives in its November number, 1887, a number of responses to letters sent out to the "leading physicians of Texas," asking for their treatment of dysentery.

Dr. C. H. Wilkinson, of Galveston, says the following is a most excellent remedy in the usual forms of dysentery seen in his city:

℞. Morph. sulphate.............................gr. $\frac{1}{3}$,

Saturat. solut. hydronaphthol..................℥ j.

M. S.: Inject three or four times a day as needed.

The ipecac plan has not proved satisfactory in his hands.

Dr. T. C. Osborne, of Cleburne, after many years of familiarity with dysentery, cleanses the intestinal canal, and then gives at bed-time, to adults; Ipecac powder, ℈ j, wetted with laudanum.ʒ j, and just enough water to make the dose easily swallowed, and then remains two hours with the patient to prevent him drinking any water. Repeat at end of twenty-four hours, *if needed.* Rarely requires more than the third repetition. If there is much hæmorrhage, he relies on ergotine rather than astringents.

Dr. F. B. Kingley, of San Antonio, has found cocaine alone or with other remedies useful. He has no routine.

Dr. S. Eagon, of Dallas, first evacuates the bowels with calomel, gr. ij, and then Rochelle salts; and then relies on the opium—doses and preparations to suit the patient. Ipecac treatment is sometimes effective, but as a rule is not well tolerated, even when associated with an opiate.

Dr. E. H. Ward, of Waxahachie, gives hypodermic injection over the stomach. In half an hour, administer full doses of ipecac—say ʒ ss. But this treatment is of little value beyond the early stage. Later on, he gives copious enemata of hot water, followed by 25 per cent. solution, in hot water, of fluid extract f hydrastis. After injections, opium suppository. Cocaine over stomach helps to relieve irritable stomach, etc.

Dr. A. C. Graham, of Dallas, in acute cases, after Rochelle salts, alternates with Batley's sedative and camphor waters. When tongue is much coated, give small doses of calomel, if needed. When becoming chronic, give small doses of corrosive sublimate or large doses of French bismuth.

Dr. John Rodman, of Abeline, says when the disease tends to chronicity, he combines aromatic sulphuric acid with Epsom salts—small doses.—*Virginia Medical Monthly.*

**Antipyretics.**—A writer in *Medical Waif* says: "Antifebrin, the latest, is decidedly the best antipyretic. It rapidly and surely reduces the temperature, and that too, without collapse, or any dangerous or alarming symptoms. It can be given to the infant as safely as to the adult. Its effects last from ten to twenty hours. In fact with attention, the temperature can almost invariably be kept at a point below the danger line. It is cheap, costing between forty and fifty cents an ounce. The dose is 7 or 8 grains to an adult, and 1 grain to a child a year and a half or two years old.

"I verily believe that with antifebrin to hold the temperature down, the mortality rate of typhoid and malarial fevers can be lessened fully one-half, and acute inflammatory diseases can have their mortality rate likewise diminished by its proper and descriminate use."

**Avulsion of Ingrowing Nail Under Hypnotism.**—Dr. S. L. Trivus, of St. Petersburg, relates (*Vratch*, No. 31, 1887, p. 607) a case in which he removed an ingrowing nail from the great toe of a cook after a hypnotic state had been induced in the patient. The operation lasted about twenty minutes. At first the woman occasionally moved her foot about, but when the author had suggested to her that no more pain was to be inflicted, and that the foot must be kept at rest, she sat quiet till the matrix of the toe was incised. At that point of the operation she shrieked out, and, when questioned about the cause, stated that a "dog had just bitten her." After applying the dressing, Dr. Trivus woke her up, and asked whether she would consent to the operation. She hesitated a little, and then said, "Yes, go on." On his pointing to her bandaged foot, however, she at once guessed that all was over already, and burst into laughter followed by hysterical sobs. No pain was felt till the next day, when she became somewhat lame. Dr. Trivus also relates an instructive case in which a young man 25, after having been hypnotized for the sake of experiment, and subsequently awakened, could not return to his normal state for several hours; when left alone, he was seized with a kind of hypnosis, and on his way home fell into a deep swoon in the street.—*British Medical Journal.*

**Painless Destruction of Nævi.**—A. B., aged 2, suffering from a nævus the size of a shilling, behind the right ear, was on May 12th, 1887, treated by me in the following manner for its removal. Having first painted the healthy skin around the circumference of the nævus, for about half an inch, with a coating of collodion flexile, I applied a thick layer of four per cent. solution corrosive sublimate in collodion over the nævus. On the 25th, when I removed the collodion, the nævus had entirely disappeared, and nothing remained but a small scab. Dr. Boing was the first to suggest this method of treatment, and my object in publishing this case is to draw attention to so simple, satisfactory, and painless a method of treatment.—*British Medical Journal.*

Dr. Ady, of Iowa, reports (*Medical and Surgical Reporter*) a case of recovery from acute peritonitis in which he gave one grain of morphia sulphate every half hour for twelve consecutive days, previous to which she had been kept under the influence of the drug for thirteen days.

**Pyridin in Asthma**.—In the course of former experiments Renzi observed that besides lessening the number of respirations, pyridin also increased the energy of the heart's systole. He therefore tested it in severe cases of heart disease, two of aortic insufficiency, one case of pericarditis, one of parenchymatous nephritis with weakened heart, and one case of mitral stenosis.

He first gave the pyridin in doses of from 6 to 10 drops, diluted with 2 or 3 drachms of water, and gradually increased the dose to 25 drops. In the cases of nephritis and mitral stenosis there was no improvement, but in the others there was a strengthening of the systolic impulse, and the number of beats was lessened. The blood-pressure was increased. A systolic action was allayed more readily by pyridin than by digitalis, and it has no cumulative effects. Angina pectoris, that often complicates such cases, was more benefited by pyridin than by anything else.—*Centralbl. fur klinische Medicin*, *No. 46*, *1887*.

**Castration in Epilepsy**.—In the *Berliner klin. Wochenschr.*, No. 3, 1887, J. Schramm reports two cases in which he performed castration for epilepsy. In the first case the ovaries were healthy, and the operation was done on account of the coincidence between the epileptic attacks and menstruation; in the second case the ovaries were cystic, but the connection between the epileptic seizures and menstruation was not so positive. Immediately after the operation repeated convulsions occurred, but subsequently ceased entirely. In the first case there was no return of the disease within eighteen months; in the second, within a year after the operation.—*Medical and Surgical Reporter*.

**A New York Wine Merchant** is given credit of having said: "They make wine nowadays without a particle of grape juice in it. We have just received from Portugal a proposition to supply us with a secret coloring matter. The proposition has it all figured out for us to show that with any amount of their stuff, costing $13 in our money, we can turn 1650 gallons of white wine into claret. They send a sample with the offer. Just so much of it as could be taken upon the point of a knife-blade turned a glass of water in an instant into the loveliest claret-colored liquid you ever saw."—*National Druggist*.

**Guaiac Tinct. in Acute Tonsillitis**.—A writer in *British Medical Journal* says of Tinct. Guaiac in Tonsillitis : "One ought to be able to abort about nine out of every ten cases, if seen in first forty-eight hours." His dose is small—two to five drops—and repeated every hour or two ; if fever runs high, he combines it with aconite tincture and some cardiac stimulant, preferably ammonia carbonate. In his communication the Doctor confines himself to its use in infants only, which agrees, in the main, with our experience, yet the remedy should not be confined to children, as equally good results follow, whatever may be the age, the dose being proportioned to the age and general temperament of the patient. Try it!

